# Functional outcomes after primary vs delayed robot-assisted radical prostatectomy following active surveillance

**DOI:** 10.1093/jncics/pkaf020

**Published:** 2025-02-06

**Authors:** Christian Corsini, Pietro Scilipoti, Andri Wilberg Orrason, Rolf Gedeborg, Marcus Westerberg, Pär Stattin

**Affiliations:** Department of Surgical Sciences, Uppsala University, Uppsala, Sweden; Division of Experimental Oncology/Unit of Urology, URI Institution: IRCCS San Raffaele Hospital, Milan, Italy; Department of Surgical Sciences, Uppsala University, Uppsala, Sweden; Division of Experimental Oncology/Unit of Urology, URI Institution: IRCCS San Raffaele Hospital, Milan, Italy; Department of Surgical Sciences, Uppsala University, Uppsala, Sweden; Department of Surgical Sciences, Uppsala University, Uppsala, Sweden; Department of Surgical Sciences, Uppsala University, Uppsala, Sweden; Department of Surgical Sciences, Uppsala University, Uppsala, Sweden

## Abstract

**Background:**

It is unknown if a period of active surveillance before prostatectomy for prostate cancer (PCa) worsens functional outcomes. The aim of this study was to compare functional outcomes after primary vs delayed robot-assisted radical prostatectomy.

**Methods:**

We included men registered in the National Prostate Cancer Register of Sweden with low and favorable intermediate-risk PCa who underwent robot-assisted prostatectomy in 2018-2020 and had filled a questionnaire on patient-reported outcome measures. Multivariable logistic regression analysis was used to compare the functional outcomes of primary and delayed prostatectomy.

**Results:**

2571 men underwent primary, and 921 men underwent delayed prostatectomy. Delayed prostatectomy was not associated with reduced overall quality of life (adjusted Odds Ratio [OR] 1.04; 95% confidence interval [CI] 0.71-1.55) or erectile dysfunction (adjusted OR 0.90, 95% CI 0.69-1.22). Urinary incontinence was slightly more common after delayed prostatectomy (15% vs 11%; adjusted OR 1.38, 95% CI 0.91-2.01). There were weak associations between time to prostatectomy and urinary symptoms and bother, with a 3% annual increase in the risk for urinary incontinence (adjusted OR 1.03; 95% CI 0.94-1.13).

**Conclusion:**

These results suggest that a period on active surveillance before robot-assisted radical prostatectomy has little detrimental effect on functional outcomes. Since only around half of men on active surveillance will transit to prostatectomy, these outcomes represent a worst-case scenario for men who start active surveillance. These results support the use of active surveillance for men with low-risk and favorable intermediate-risk PCa.

## Graphical Abstract

**Graphical Abstract:**
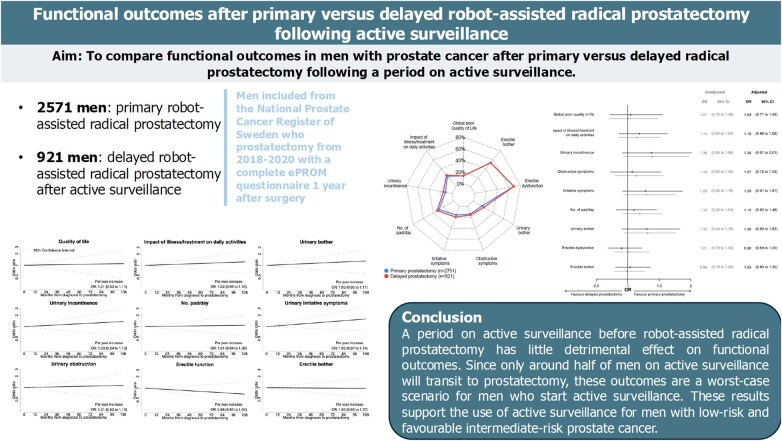

## Introduction

The aim of active surveillance in men with prostate cancer (PCa) is to avoid adverse effects of radical treatment without jeopardizing oncological outcomes.^1-4^ Nevertheless, according to results from the ProtecT trial, approximately half of men assigned to active surveillance underwent delayed radical treatment within 15 years of follow-up.^2^ Similarly, in Sweden around 30% of men on active surveillance received delayed radical treatment within 4 years after diagnosis, which is in accordance with other studies with longer follow-up.^4-6^

Adverse effects of radical robot-assisted prostatectomy on erectile function and urinary continence are well documented.^7-9^ In a recent population-based study in Sweden of men who had undergone robot-assisted prostatectomy in 2017-2019, 14% of men reported urinary incontinence in an electronic patient-reported outcome measures questionnaire (*e*PROM).^10^ In men on active surveillance, continence and sexual function decline over time due to aging, or disease progression.^11^^,^^12^ Men on active surveillance were reported to have similar overall quality of life as men who have undergone radical treatment in a recent review.^13^ Few studies have compared quality of life and functional outcomes after primary vs delayed radical prostatectomy, and they have been limited by small sample size or suboptimal design and often do not include men on a previous active surveillance protocol.^14-19^

The aim of this study was to compare quality of life and functional outcomes in men with low and favorable intermediate-risk PCa after robot-assisted prostatectomy with results after a delayed robot-assisted prostatectomy following a period of active surveillance.

## Methods

### Data sources

The National Prostate Cancer Register of Sweden (NPCR) captures 98% of all incident PCa cases compared to the Cancer Register to which reporting is mandated by law.^20^ NPCR is a clinical cancer register with the primary aim to ensure high quality of care for men with PCa and to monitor adherence to national guidelines.^21-23^ Pre, intra, and postoperative data on men treated with radical prostatectomy are recorded in NPCR. In Prostate Cancer data Base Sweden (PCBase), NPCR has been enriched by linkages to other health care registers and demographic databases including The Patient Register, The Longitudinal integrated database for health insurance and labor market studies, a socioeconomic database, and The Prescribed Drug Register.

### Study population

We included men from NPCR with a diagnosis of PCa who would be eligible for active surveillance according to Swedish guidelines,^21-23^ ie, had clinical stage T1-T2, Gleason score less than or equal to 7 (3 + 4) on diagnostic prostate biopsy, prostate-specific antigen (PSA) levels less than or equal to 20 ng/mL, no lymph node (N0 or Nx) or distant metastases (M0 or Mx) who underwent prostatectomy between January 2018 and December 2020, and who had filled a self-administered online “electronic” patient-reported outcome measures questionnaire (*e*PROM—NPCR version 2016-09) distributed by third party 1 year after date of surgery as described in previous studies.^10^ Robot-assisted prostatectomy was performed in 31 hospitals in Sweden during the study period and NPCR captured all robot-assisted prostatectomies performed at these hospitals. Primary prostatectomy was defined as prostatectomy performed within 12 months from diagnosis, and delayed prostatectomy was defined as prostatectomy performed after a minimum of 12 months of active surveillance initiated between 2010 and 2020.^24^ Comorbidity at date of prostatectomy was assessed by use of the Drug Comorbidity Index and the Multidimensional Comorbidity Index.^25^^,^^26^ Life expectancy was calculated from age and both comorbidity indices.^27^

### Registration of patient reported outcome measures (ePROM) in NPCR

The *e*PROM in NPCR consists of 35 questions including general health, lower urinary tract symptoms, bowel symptoms, and sexual function, designed by the NPCR steering group in collaboration with quality of life experts. The baseline *e*PROM is distributed by the treating department before robot-assisted radical prostatectomy, but not before AS. One year after prostatectomy, The Mid-Sweden Regional Cancer Centre, ie, a third party without any association with the treating department distributes a letter with an invitation to fill an *e*PROM online to all men registered with a prostatectomy in NPCR, including men who have not completed the baseline *e*PROM.^10^

### Outcomes

Symptoms were assessed by one question per domain except erectile dysfunction that was assessed by use of the International Index of Erectile Function-5 scale. A detailed description of the questions used and the dichotomization of the response to each question is provided in Supplementary Methods S1.

### Statistical analysis

Missing data on preoperative PSA, clinical and pathological T stage, Gleason score at prostatectomy, prostate volume, PSA density, educational level, and civil status were imputed using multiple imputation with 5 imputations and 10 iterations.^28^ Primary vs delayed prostatectomy were compared using logistic regression analysis with primary prostatectomy as reference. Odds ratios (ORs) were adjusted for age at surgery, preoperative PSA, prostate volume, Gleason score at prostatectomy, pathological T stage, comorbidity (Drug Comorbidity Index and the Multidimensional Comorbidity Index) at date of surgery, educational level, and civil status, by use of stabilized inverse probability of treatment weighting.^29^ We used thin plate regression splines for all continuous variables including age at surgery. Weights were assessed by computing the standardized mean difference for each confounder. 95% confidence intervals (CI) were computed by use of bootstrapping with 1000 resamplings with the boot multiple imputation percentile method.^30-33^

An analysis of the subgroup of men who had filled *e*PROM prior to prostatectomy was performed and in this analysis with adjustment for erectile dysfunction before prostatectomy. We also estimated the association between time from diagnosis to prostatectomy as a continuous variable and each outcome. In this analysis, adjusted ORs were computed to assess the annual change in the outcomes between the date of diagnosis and date of prostatectomy. We used a multivariable logistic regression model, adjusting for the same variables as previously listed, with thin plate regression splines applied to all continuous variables, including time to prostatectomy.^33^ Finally, we compared clinical characteristics between responders and non-responders of the questionnaire.

All tests were two-sided, and the statistical significance level was determined at *P* < .05. Statistical analyses were performed with R version 3.5.3 (R Foundation). The Swedish Ethical Review Authority approved of the study. The requirement for informed consent was waived by this authority.

## Results

### Cancer characteristics at diagnosis and at robot-assisted radical prostatectomy

The study included 3672 men who underwent robot-assisted radical prostatectomy and had filled an *e*PROM 1 year after surgery, of whom 2751 underwent primary prostatectomy and 921 underwent delayed prostatectomy after a period of active surveillance (Table 1, Figure S1). Median time on active surveillance before prostatectomy was 3 years, with 37% followed for over 4 years, and 11% on surveillance after 6 years (Table 2).

**Table 1.** Baseline and perioperative characteristics at date of diagnosis of men with low-risk and favorable intermediate-risk prostate cancer in the Prostate Cancer data Base Sweden 5.0 who in 2018-2020 underwent primary or delayed robot-assisted radical prostatectomy, after a period of active surveillance.

Characteristic	Primary prostatectomy No. (%)	Delayed prostatectomy No. (%)
**No. of men**	2751 (100)	921 (100)
**Age, years**		
Median (Interquartile range [IQR])	64 (59-69)	63 (59-67)
<60	728 (26)	272 (30)
60-64	661 (24)	264 (29)
65-70	788 (29)	284 (31)
71-75	574 (21)	101 (11)
**Educational level** ^a^		
High	1138 (41)	388 (42)
Intermediate	1202 (44)	378 (41)
Low	404 (15)	154 (17)
Missing	7	1
**Civil status**		
Married	2354 (86)	763 (83)
Divorced	340 (12)	135 (15)
Widower	57 (2)	23 (2)
**Life expectancy, years**		
Median (IQR)	20 (17-25)	21 (18-25)
**PSA, ng/mL** ^b^		
Median (IQR)	5.7 (4.1-8.1)	5.2 (4.0-7.0)
**Prostate volume, cc**		
Median (IQR)	35 (28-46)	36 (29-48)
Missing	68	31
**PSA density, ng/mL/cc**		
Median (IQR)	0.16 (0.11-0.23)	0.14 (0.11-0.19)
Missing	68	31
**Gleason score at diagnostic biopsy**		
Gleason 6	654 (24)	821 (89)
Gleason 7 (3 + 4)	2097 (76)	100 (11)
**Clinical T stage**		
T1	1883 (68)	794 (86)
T2	868 (32)	125 (14)
Missing	2	0

a Years of schooling: low, less than 10 years; intermediate, 10–12 years; and high, more than 12 years.

b PSA = prostate-specific antigen.

**Table 2.** Baseline and perioperative characteristics at date of robot-assisted radical prostatectomy of men with low-risk and favorable intermediate-risk prostate cancer in the Prostate Cancer data Base Sweden 5.0 who in 2018-2020 underwent primary or delayed robot-assisted radical prostatectomy, after a period of active surveillance.

Characteristic	Primary prostatectomy No. (%)	Delayed prostatectomy No. (%)
**No. of patients**	2751 (100)	921 (100)
**Age, years**		
Median (IQR)	65 (60-69)	67 (62-71)
<60	675 (25)	122 (13)
60-64	651 (24)	219 (24)
65-70	768 (28)	256 (28)
71-75	657 (24)	324 (35)
**PSA, ng/mL** ^a^		
Median (IQR)	5.9 (4.2-8.5)	7.1 (4.9-10.0)
Missing	5	17
**Gleason score in prostatectomy specimen**		
Gleason 6	500 (18%)	404 (46%)
Gleason 7 (3 + 4)	1750 (64%)	327 (37%)
Gleason 7 (4 + 3)	453 (17%)	123 (14%)
Gleason 8-9-10	14 (1)	31 (3)
Missing	34	36
**Pathological T stage**		
T2	1979 (73)	639 (70)
T3	739 (22)	269 (30)
Missing	33	13
**Time from diagnosis to RP, months** ^c^		
Median (IQR)	3 (2-5)	39 (24-57)
≤12	2751 (100)	–
13-24	–	230 (25)
25-48	–	352 (38)
49-72	–	228 (25)
>72	–	111 (12)
**Nerve-sparing RARP** ^b^		
No	308 (11)	120 (13)
Unilateral	625 (23)	179 (19)
Bilateral	1,818 (66)	622 (68)
**Surgical margins**		
Negative	2007 (74)	675 (74)
Positive	711 (26)	233 (25)
Unclear	12 (0)	4 (0)
Missing	21	9

a PSA = prostate-specific antigen.

b RARP = robot-assisted radical prostatectomy.

c RP = radical prostatectomy.

Before imputation, 187 men (5%) had missing data in at least 1 variable, with similar proportions in the 2 groups for all covariates (Tables 1 and 2).

Gleason score 6 at diagnosis was less common in men who underwent primary prostatectomy (24%, *n* = 654) compared to men who underwent delayed prostatectomy (89% *n* = 821) (Table 1). The difference in Gleason score was smaller at the examination of the surgical specimen with Gleason score 6 less common after primary prostatectomy (18%, *n* = 500) compared to delayed prostatectomy (46%, *n* = 404). (Table 2) Pathological T3 stage was found in 22% after primary prostatectomy and 30% after delayed prostatectomy.

### Overall quality of life

Quality of life data collected before prostatectomy was available for 2201 men (60%) and among these men the proportion with poor quality of life was similar in the two groups (5.2% vs 6.5% for primary vs delayed prostatectomy).

**Figure 1.**
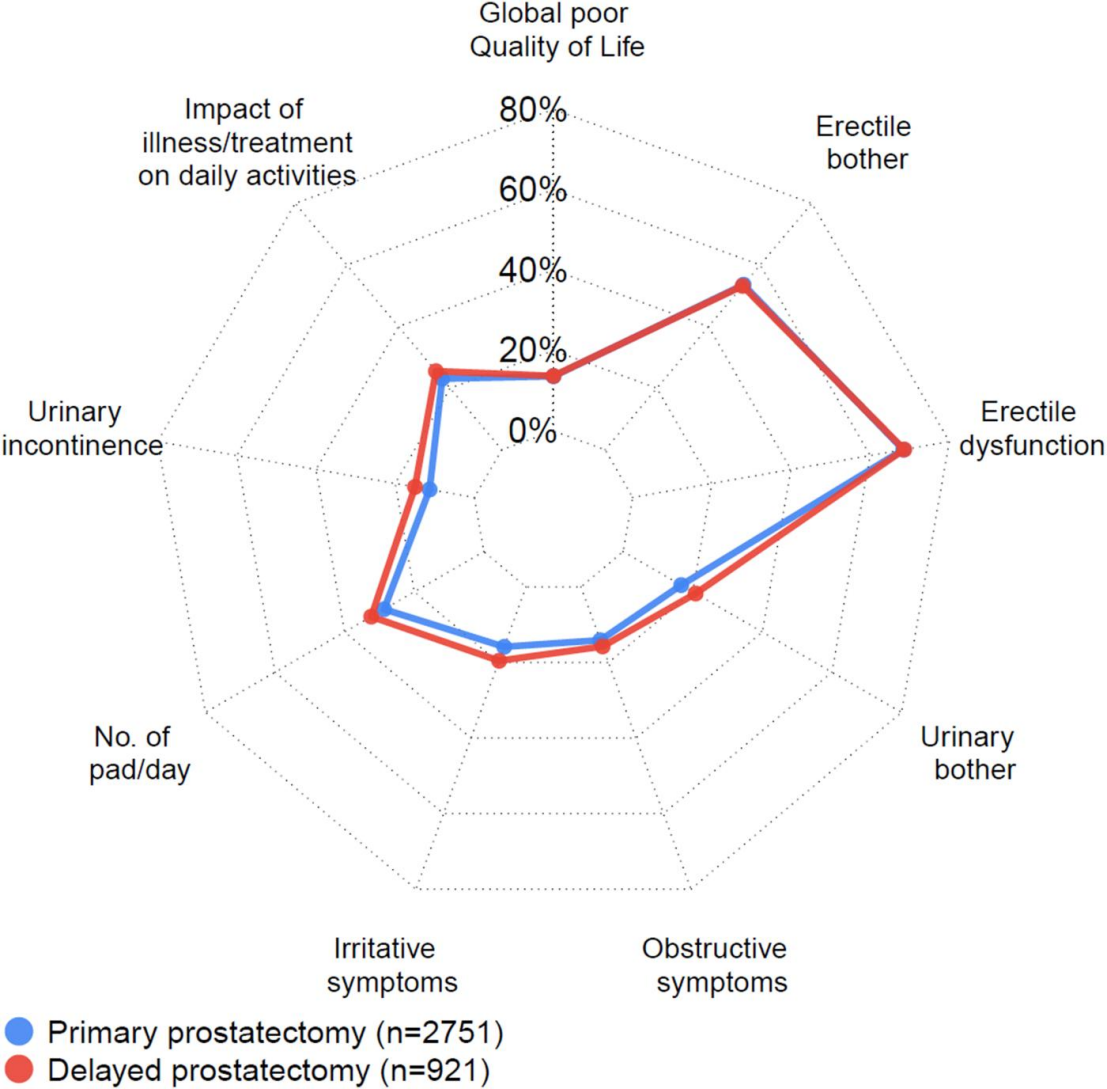
Quality of life and functional outcomes after primary vs delayed robot-assisted radical prostatectomy.

**Figure 2.**
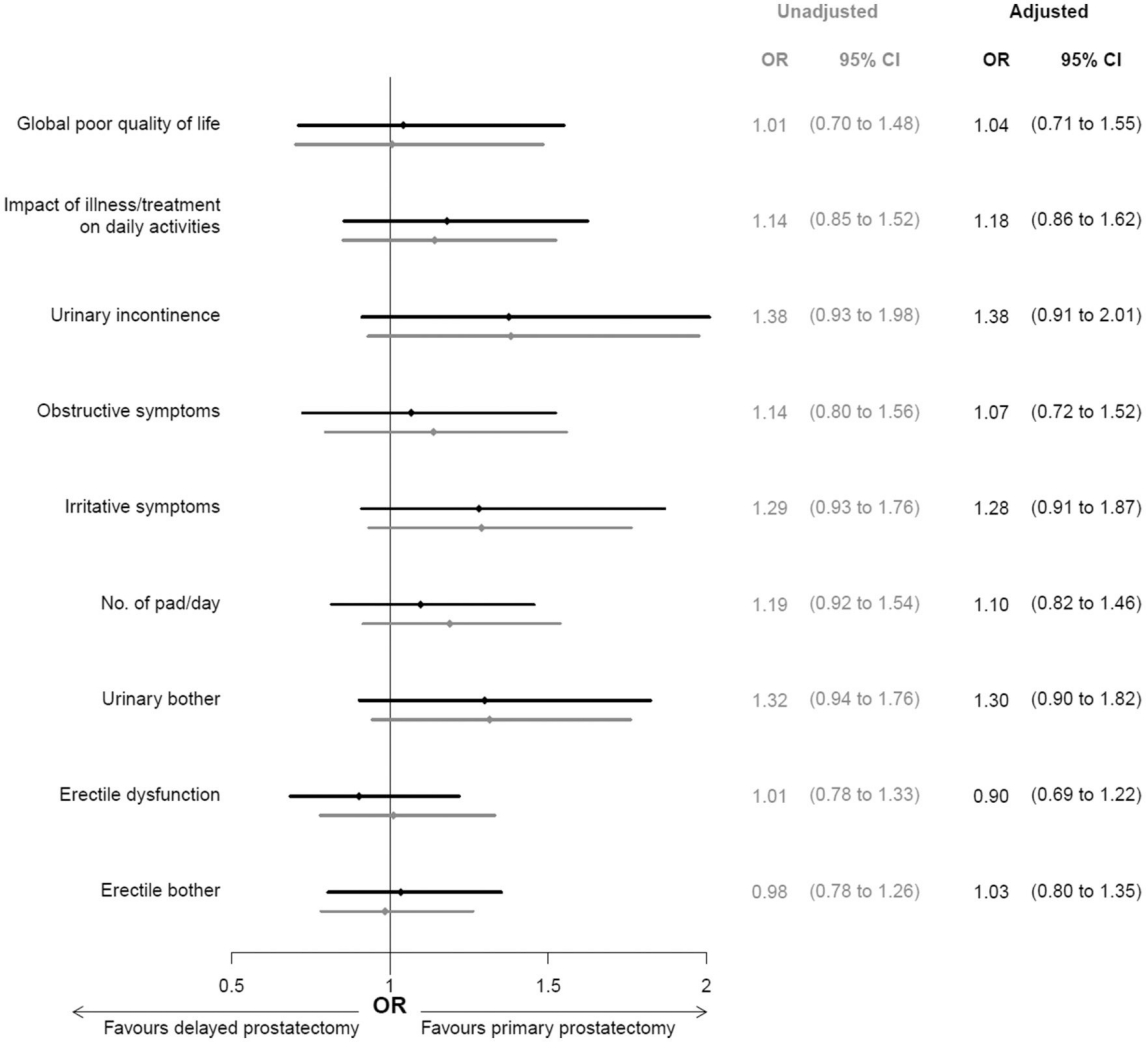
Difference in quality of life and functional outcomes after primary (reference) vs delayed robot-assisted radical prostatectomy. ORs were calculated with logistic regression with primary prostatectomy as reference and adjusted for socioeconomic and clinical characteristics: age at time of surgery, comorbidity status at surgery, PSA at surgery, pathological Gleason score and pathological T stage, prostate volume and civil status and educational level. OR = odds ratio, CI = confidence interval (gray color for unadjusted analysis, black for adjusted analysis), PSA = prostate-specific antigen.

Overall quality of life did not differ after primary and delayed prostatectomy (Figures 1 and 2). Poor overall quality of life was reported by 14% of men both after primary prostatectomy and delayed prostatectomy (OR 1.04, 95% CI 0.71-1.55). The proportions of men who reported an impact of illness/treatment on daily activities were 23% after primary prostatectomy and 26% after delayed prostatectomy and adjustment for covariates had little impact on this association (OR 1.18, 95% CI 0.86-1.62).

### Urinary symptoms and bother

There was a slightly lower proportion of men who had urinary symptoms or bother after primary compared to delayed prostatectomy; urinary incontinence (11% vs 15%), number of pads (27% vs 32%), urinary irritative symptoms (16% vs 20%), high urinary bother (17% vs 21%), and obstructive symptoms (14% vs 16%). After adjustment for potential confounders, there was a slightly higher risk for urinary incontinence (OR 1.38, 95% CI 0.91-2.01) and urinary irritative symptoms (OR 1.28, 95% CI 0.91-1.87) after delayed prostatectomy (Figure 2).

**Figure 3.**
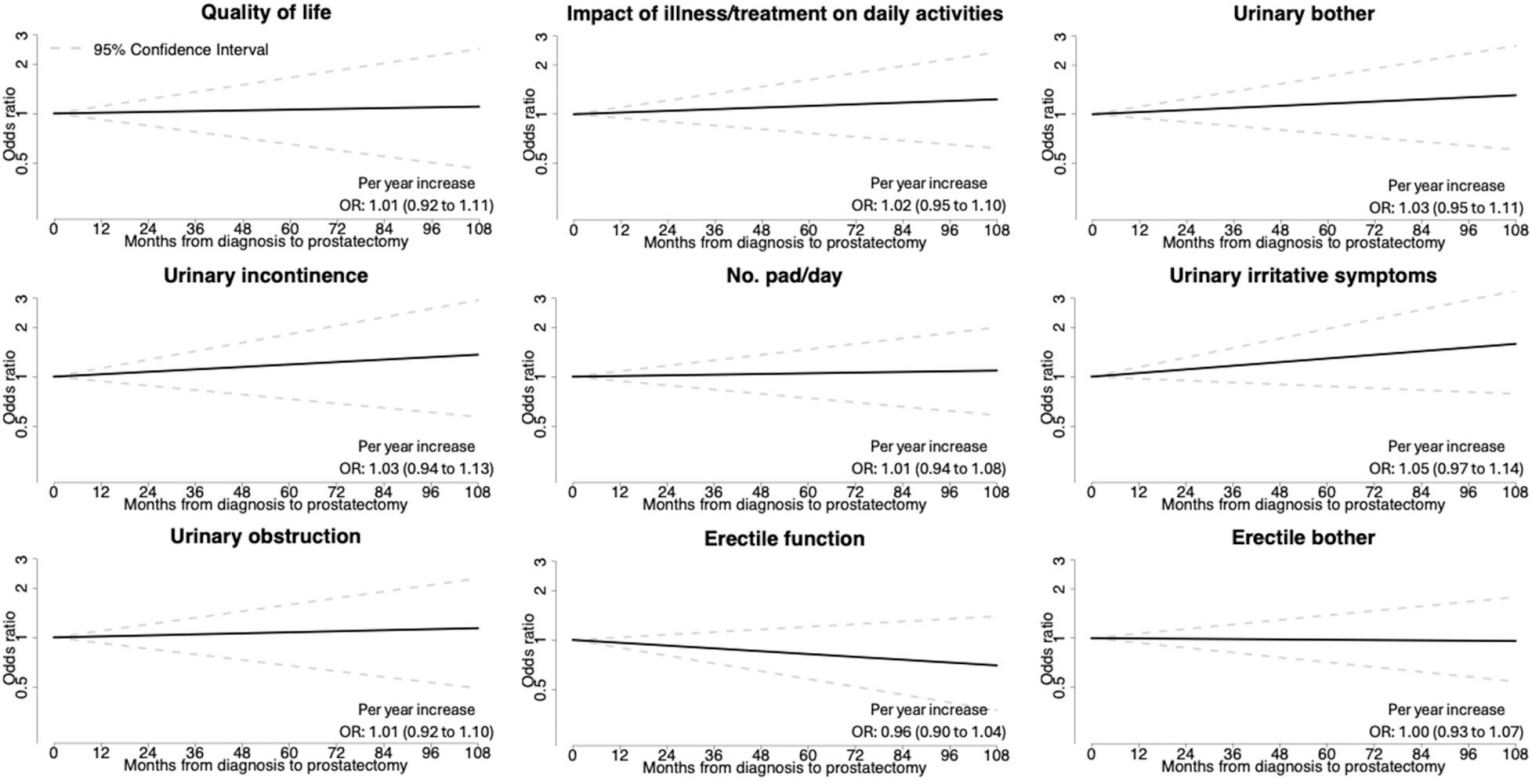
Functional outcome recovery expressed in terms of ORs as a function of the time between diagnosis and robot-assisted radical prostatectomy. The time-dependent OR was estimated using a multivariable logistic regression model adjusting for age at time of surgery, comorbidity indices at surgery, PSA at surgery, Gleason score in surgical specimen and pathological T stage, prostate volume, civil status, and educational level. OR = odds ratio.

In analysis of the association between time to prostatectomy as continuous variable and urinary symptoms there was a 1% annual increase in risk of number of pads needed (OR 1.01, 95% CI 0.94-1.08), a 3% annual increase in risk of urinary incontinence (OR 1.03, 95% CI 0.94-1.13), and a 5% annual increase in risk of urinary irritative symptoms (OR 1.05, 95% CI 0.97-1.14) (Figure 3).

### Erectile dysfunction and bother

The proportions of men with erectile dysfunction (68% vs 69%) or erectile bother (54% vs 53%) were similar after primary vs delayed prostatectomy (Figure 1). After adjustment for covariates the risk for erectile dysfunction was not increased after delayed prostatectomy (OR 0.90, 95% CI 0.69-1.22) (Figure 2).

In the analysis of 1455 men who had also filled a baseline *e*PROM shortly before date of prostatectomy, erectile dysfunction before prostatectomy was reported by 43% of men in the primary group and 48% in the delayed group. In an analysis adjusted for baseline erectile dysfunction, a similar result as in the full study population of risk of erectile dysfunction after delayed prostatectomy vs primary prostatectomy was observed (OR 0.95; 95% CI 0.82-1.09) (Table S1).

### Analysis of non-responders to ePROM

There were 1994 (35%) men who matched the inclusion criteria for the study but had not filled the *e*PROM. Non-responders were similar to responders in all baseline characteristics except high educational level that was more common in responders than non-responders (42% vs 31%) (Table S2).

## Discussion

In this large population-based study of men with low-risk and favorable intermediate-risk PCa who underwent primary or delayed prostatectomy after a period on active surveillance before surgery, *e*PROM data 1 year after surgery showed little detrimental effect on quality of life and functional outcomes of the delay. In men who underwent delayed prostatectomy, there was no increase in risk of poor overall quality of life or erectile dysfunction and the increase in risk of urinary problems was weak and therefore sensitive to remaining bias, despite the large sample size and the rather large time range on active surveillance.

### Strengths and limitations

Our study has several strengths. NPCR has a 98% capture rate of all men with PCa compared to the Cancer Register to which registration is mandated by law.^20^ Similarly, capture rate of prostatectomy performed in Sweden was virtually complete in NPCR (98%) in 2018-2020, and our study included men who underwent prostatectomy at all Swedish hospital where prostatectomy was performed. In addition to data in NPCR, we had access to data on comorbidity and socioeconomic factors from other nationwide health care registers and demographic databases with high data quality. Furthermore, there were only small differences in baseline characteristics between responders and non-responders to the *e*PROM, except for a slightly higher proportion of men with high educational level among responders, indicating no strong selection bias related to the response rate.

Around 65% of men in Sweden who underwent robot-assisted radical prostatectomy in 2018-2020 had filled an *e*PROM 1 year after surgery. This *e*PROM was created by NPCR to suit routine quality assurance of all men who undergo radical prostatectomy in Sweden and was quite short to optimize the response rate. The same *e*PROM was distributed to all men in the study 1 year after surgery by an independent third party, without any association with the treating department, minimizing courtesy bias (eg, responder wanting to please the treating department provider).^34^

Erectile dysfunction was assessed by use of the validated and commonly used International Index of Erectile Function-5 scale.^35^^,^^36^ The lack of preoperative data for a majority of men hampers the interpretation of data on erectile function. However, in a subgroup analysis of men who had responded both to baseline and 1-year *e*PROM there was no difference in erectile function after primary and delayed prostatectomy when adjusted for erectile function at baseline. Concerning urinary problems, missing data on baseline function was less of a problem since urinary incontinence before prostatectomy is rare. In a recent NPCR study, *e*PROMs collected before prostatectomy showed a frequency of 2% of urinary incontinence.^10^ There was also reasonable consistency between different measures of urinary symptoms and bother in support of that our results are valid. Furthermore, we did not have data on prostate biopsies performed during AS, which could have provided insights into their potential impact on functional outcomes.

Of note, the *e*PROM questionnaire was not administered to men before start of active surveillance. Finally, due to the inherent limitations of the study observational design, there were differences in baseline characteristics, eg, lower rate of pathological T3 after primary vs delayed prostatectomy. We included adjustment for factors that could have influenced risk in multivariable analyses, but unmeasured differences between the groups could still have influenced our risk estimates.

### Previous studies

No randomized controlled trial has been conducted on this topic to the best of our knowledge, and we argue that it is unlikely that such a study will be performed. Previous observational studies on functional outcomes after primary vs delayed prostatectomy have been hampered by small numbers and/or suboptimal design.^14-17^ In a Canadian single institution study of 48 men who underwent prostatectomy between 1992 and 2009 after a period of active surveillance, risk of urinary incontinence was similar in men treated with primary and delayed prostatectomy.^14^ Three other studies investigated the association between time from diagnosis to prostatectomy and functional outcomes without a specific active surveillance group.^15-17^ In an Italian single center study, 22 men underwent prostatectomy after more than 12 months from diagnosis with no increased risk of erectile dysfunction compared to those who underwent prostatectomy within 6 months.^15^ In a study using the Surveillance, Epidemiology, and End Results Program database with administrative data on diagnoses and procedures (ie, no data on PROM), including men who underwent prostatectomy from 1995 to 2005, those who underwent prostatectomy after three months from diagnosis had a higher risk of erectile dysfunction and urinary incontinence.^16^ A propensity-matched United States study of 181 men who had primary or delayed prostatectomy after an “assumed period of active surveillance” showed no difference in erectile function or urinary continence.^17^

Another retrospective single-center study compared 363 men who underwent primary prostatectomy with 29 men who underwent delayed prostatectomy in 2007-2012. Patients in the delayed group had better erectile function and less urinary symptoms at baseline than men who underwent primary prostatectomy and these differences remained after surgery.^18^

### Interpretation and generalizability

To the best of our knowledge, this is by far the largest study of functional outcomes after primary vs delayed prostatectomy in men with low-risk and favorable intermediate-risk PCa, ie, in men for whom active surveillance is indicated. However, despite our large sample size it was difficult to estimate the weak associations with high precision since weak associations in self-reported outcomes are sensitive to selection bias and misclassification so consequently our results need to be interpreted with caution. Importantly, our results should be interpreted in the context of how active surveillance is performed. There are inherent biases both in the selection of active surveillance and in the selection of transit from active surveillance to prostatectomy. A transition to radical treatment should be performed if and when progression occurs, and this transfer is made by half or less than half of men who start active surveillance.^2^^,^^4^ There is a selection of men with favorable cancer features to active surveillance and a selection of men with adverse features who transit from active surveillance and the effect of these selections are unlikely to be fully compensated by the adjustments in our analyses. Our results should be viewed as a “worst case scenario” for functional outcomes for men who start active surveillance since half or more of men on active surveillance will not transit to radical treatment and will be spared from its adverse effects. We argue that our estimates are likely to be applicable to men in other settings since the indications for active surveillance and transit to radical treatment are essentially similar in guidelines issued by European Association of Urology and American Urological Association.^37^^,^^38^

In conclusion, in this large population-based register study there was no difference in overall quality of life and erectile function in men with low-risk and favorable intermediate-risk PCa after primary vs delayed robot-assisted radical prostatectomy assessed by *e*PROM one year after surgery. There was an increase in urinary symptoms and bother after delayed radical prostatectomy, however, the association was weak and therefore sensitive to remaining bias. Our results suggest that a period of active surveillance before radical prostatectomy has little detrimental effect on functional outcomes. Since only around half of men on active surveillance transit to prostatectomy, the results after delayed treatment represent a worst-case scenario of functional outcomes for men on active surveillance. These results support the use of active surveillance for men with low-risk and favorable intermediate-risk PCa.

## Supplementary Material

pkaf020_Supplementary_Data

## Data Availability

Data used in this study was extracted from the Prostate Cancer data Base Sweden (PCBaSe), which is based on the National Prostate Cancer Register (NPCR) of Sweden. The data cannot be shared publicly because the individual-level data contain potentially identifying and sensitive patient information and cannot be published due to legislation and ethical approval (https://etikprovningsmyndigheten.se). Use of the data from national health-data registers is further restricted by the Swedish Board of Health and Welfare (https://www.socialstyrelsen.se/en/) and Statistics Sweden (https://www.scb.se/en/) which are Government Agencies providing access to the linked healthcare registers. Pseudoanonymized data can be shared on a remote server without possibility of exporting data individuals on reasonable request in an application made to the steering group of PCBaSe. For detailed information, please see www.npcr.se/in-english, where registration forms, manuals, and annual reports from NPCR are also available alongside a full list of publications from PCBaSe. The statistical program code used for the present study analyses can be provided by christian.corsini@hsr.it.

## References

[1] Willemse PPM , Davis NF, Grivas N, et al Systematic review of active surveillance for clinically localised prostate cancer to develop recommendations regarding inclusion of intermediate-risk disease, biopsy characteristics at inclusion and monitoring, and surveillance repeat biopsy strategy. Eur Urol. 2022;81:337-346. 10.1016/j.eururo.2021.12.007 34980492

[2] Hamdy FC , Donovan JL, Lane JA, et al Fifteen-year outcomes after monitoring, surgery, or radiotherapy for prostate cancer. N Engl J Med. 2023;388:1547-1558. 10.1056/nejmoa2214122 36912538

[3] Loeb S , Folkvaljon Y, Bratt O, Robinson D, Stattin P. Defining intermediate risk prostate cancer suitable for active surveillance. J Urol. 2019;201:292-299. 10.1016/j.juro.2018.09.042 30240688

[4] Stattin P , Holmberg E, Johansson JE, Holmberg L, Adolfsson J, Hugosson J; National Prostate Cancer Register (NPCR) of Sweden. Outcomes in localized prostate cancer: National Prostate Cancer Register of Sweden follow-up study. J Natl Cancer Inst. 2010;102:950-958. 10.1093/jnci/djq154 20562373 PMC2897875

[5] Welty CJ , Cowan JE, Nguyen H, et al Extended followup and risk factors for disease reclassification in a large active surveillance cohort for localized prostate cancer. J Urol. 2015;193:807-811. 10.1016/j.juro.2014.09.094 25261803

[6] Klotz L , Vesprini D, Sethukavalan P, et al Long-term follow-up of a large active surveillance cohort of patients with prostate cancer. J Clin Oncol. 2015;33:272-277. 10.1200/JCO.2014.55.1192 25512465

[7] Resnick MJ , Koyama T, Fan KH, et al Long-term functional outcomes after treatment for localized prostate cancer. N Engl J Med. 2013;368:436-445. 10.1056/nejmoa1209978 23363497 PMC3742365

[8] Corsini C , Bergengren O, Carlsson S, et al Patient-reported side effects 1 year after radical prostatectomy or radiotherapy for prostate cancer: a register-based nationwide study. Eur Urol Oncol. 2024;7:605-613. 10.1016/j.euo.2023.12.007 38233329 PMC11102330

[9] Nahas WC , Rodrigues GJ, Rodrigues Gonçalves FA, et al Perioperative, oncological, and functional outcomes between robot-assisted laparoscopic prostatectomy and open radical retropubic prostatectomy: a randomized clinical trial. J Urol. 2024;212:32-40. 10.1097/JU.0000000000003967 38723593

[10] Arnsrud Godtman R , Persson E, Bergengren O, et al Surgeon volume and patient-reported urinary incontinence after radical prostatectomy. Population-based register study in Sweden. Scand J Urol. 2022;56:343-350. 10.1080/21681805.2022.2119270 36068973

[11] Donovan JL , Hamdy FC, Lane JA, et al Patient-reported outcomes after monitoring, surgery, or radiotherapy for prostate cancer. N Engl J Med. 2016;375:1425-1437. 10.1056/nejmoa1606221 27626365 PMC5134995

[12] Johansson E , Steineck G, Holmberg L, et al Long-term quality-of-life outcomes after radical prostatectomy or watchful waiting: The Scandinavian Prostate Cancer Group-4 randomised trial. Lancet Oncol. 2011;12:891-899. 10.1016/S1470-2045(11)70162-0 21821474

[13] Thompson D , Bensley JG, Tempo J, et al Long-term health-related quality of life in patients on active surveillance for prostate cancer: a systematic review. Eur Urol Oncol. 2023;6:4-15. 10.1016/j.euo.2022.09.001 36156268 PMC9908828

[14] Radomski L , Gani J, Trottier G, Finelli A. Active surveillance failure for prostate cancer: does the delay in treatment increase the risk of urinary incontinence? Can J Urol. 2012;19:6287. 22704315

[15] Schifano N , Capogrosso P, Pozzi E, et al Impact of time from diagnosis to treatment on erectile function outcomes after radical prostatectomy. Andrology. 2020;8:337-341., 10.1111/andr.12699 31478610

[16] Sun M , Abdollah F, Hansen J, et al Is a treatment delay in radical prostatectomy safe in individuals with low-risk prostate cancer? J Sexual Med. 2012;9:2961-2969. 10.1111/j.1743-6109.2012.02806.x 22672479

[17] Rocco B , Sighinolfi MC, Covas Moscovas M, et al May outcomes of robotic radical prostatectomy performed after an initial surveillance strategy differ from those from immediate surgery? a propensity score-matched analysis on 362 patients undergoing surgery at a referral center. J Endourol. 2022;36:1302-1308. 10.1089/end.2021.0812 35152779

[18] Van Den Bergh RCN , De Blok W, Van Muilekom E, Tillier C, Venderbos LD, Van Der Poel HG. Impact on quality of life of radical prostatectomy after initial active surveillance: more to lose? Scand J Urol. 2014;48:367-373. 10.3109/21681805.2013.876097 24506062

[19] Chan VWS , Tan WS, Asif A, et al Effects of delayed radical prostatectomy and active surveillance on localised prostate cancer—a systematic review and meta-analysis. Cancers (Basel). 2021;13:3274. 10.3390/cancers13133274 34208888 PMC8268689

[20] Tomic K , Berglund A, Robinson D, et al Capture rate and representativity of the National Prostate Cancer Register of Sweden. Acta Oncol. 2015;54:158-163. 10.3109/0284186X.2014.939299 25034349

[21] Stranne J. 2023/2024 update of the national prostate cancer guidelines in Sweden. Scand J Urol. 2024 2024;59:210-211. 10.2340/sju.v59.42656 39714048

[22] Stattin P. How to improve cancer care by use of guidelines and quality registers. Scand J Urol. 2024;59:190-192. 10.2340/sju.v59.42272 39692279

[23] Regionala Cancercentrum i Samverkan. National Swedish guidelines on prostate cancer. Accessed January 15, 2025. https://cancercentrum.se/samverkan/cancerdiagnoser/prostata/vardprogram/

[24] Nationella prostatacancerregistret. RATTEN—interaktiv onlinerapport från NPCR. Accessed January 15, 2025. https://statistik.incanet.se/npcr/

[25] Westerberg M , Irenaeus S, Garmo H, Stattin P, Gedeborg R. Development and validation of a multidimensional diagnosis-based comorbidity index that improves prediction of death in men with prostate cancer: nationwide, population-based register study. PLoS One. 2024;19:e0296804. 10.1371/journal.pone.0296804 38236934 PMC10796041

[26] Gedeborg R , Sund M, Lambe M, et al An aggregated comorbidity measure based on history of filled drug prescriptions: development and evaluation in two separate cohorts. Epidemiology. 2021;32:607e15. 10.1097/EDE.0000000000001358 33935137

[27] Westerberg M , Ahlberg M, Wilberg Orrason A, Gedeborg R. Assessment of variability in life expectancy in older men by use of new comorbidity indices. A nationwide population-based study. Scand J Urol. 2024;59:207-209. 10.2340/sju.v59.42504 39704547

[28] van Buuren S. Flexible Imputation of Missing Data. CRC press; 2012. 10.1201/b11826

[29] Toh S , García Rodríguez LA, Hernán MA. Analyzing partially missing confounder information in comparative effectiveness and safety research of therapeutics. Pharmacoepidemiol Drug Saf. 2012;21(Suppl 2):13-20. 10.1002/pds.3248 22552975 PMC3727636

[30] Wood SN. Thin plate regression splines. J R Stat Soc Series B Stat Methodol. 2003;65. 10.1111/1467-9868.00374

[31] Wood SN. Generalized Additive Models: An Introduction with R. CRC; 2006.

[32] Wood SN. Generalized Additive Models: An Introduction with R, 2 Edition. Chapman and Hall/CR; 2017.

[33] Bartlett JW , Hughes RA. Bootstrap inference for multiple imputation under uncongeniality and misspecification. Stat Methods Med Res. 2020;29:3533-3546. 10.1177/0962280220932189 32605503 PMC7682506

[34] Hameed W , Ishaque M, Gul X, et al Does courtesy bias affect how clients report on objective and subjective measures of family planning service quality? A comparison between facility- and home-based interviews. Open Access J Contracept. 2017;9:33-43. 10.2147/oajc.s153443 29760573 PMC5937485

[35] Godtman RA , Persson E, Cazzaniga W, et al Association of surgeon and hospital volume with short-term outcomes after robotassisted radical prostatectomy: nationwide, population-based study. PLoS One 2021;16:e0253081. 10.1371/journal.pone.0253081 34138904 PMC8211177

[36] Cazzaniga W , Godtman RA, Carlsson S, et al Population-based, nationwide registration of prostatectomies in Sweden. J Surg Oncol. 2019;120:803-812. 10.1002/jso.25643 31355454 PMC6771627

[37] Eastham JA , Auffenberg GB, Barocas DA, et al Clinically localized prostate cancer: AUA/ASTRO guideline, part ii: principles of active surveillance, principles of surgery, and follow-up. J Urol. 2022;208:19-25. 10.1097/JU.0000000000002758 35536148

[38] Cornford P , van den Bergh RCN, Briers E, et al EAU-EANM-ESTRO-ESUR-ISUP-SIOG guidelines on prostate cancer—2024 update. part i: screening, diagnosis, and local treatment with curative intent. Eur Urol. 2024;86:148-163. 10.1016/j.eururo.2024.03.027 38614820

