# Supplementary material for: Functional outcomes after primary vs delayed robot-assisted radical prostatectomy following active surveillance
Source: JNCI Cancer Spectr. 2025 Feb 6;9(2):pkaf020. doi: 10.1093/jncics/pkaf020 (PMC11884805; doi:10.1093/jncics/pkaf020)
Supplement: pkaf020_Supplementary_Data [file pkaf020_supplementary_data.docx]

**Supplementary methods.** Supplementary methods to “Functional outcomes after primary versus delayed robot-assisted radical prostatectomy following active surveillance.”

**Supplementary table 1.** Multivariable logistic regression analysis after inverse probability of treatment weighting of 1455 men who had filled baseline and 1-year ePROM after robot-assisted radical prostatectomy and had complete data on baseline erectile function.

The model was adjusted for: age at surgery, multidimensional comorbidity index and Drug comorbidity index at time of surgery, pathological Gleason score and T stage, preoperative prostate-specific antigen, prostate volume at diagnosis, educational level and civil status. In addition, baseline erectile function was included.

**Supplementary table 2.** Characteristics of responders vs. non-responders to the 1-year ePROM questionnaire after primary or delayed robot-assisted radical prostatectomy.

**Supplementary figure 1.** Flow chart of study population in Prostate Cancer data Base Sweden 5.0 of men with prostate cancer who according to the National Prostate Cancer Register underwent primary or delayed robot-assisted radical prostatectomy after a period of active surveillance from 2018 to 2020.

**Supplementary methods to**

**Functional outcomes after primary versus delayed robot-assisted radical prostatectomy following active surveillance**

**Definition of outcomes**

*Urinary domain*

- Urinary incontinence: defined as “Moderately” or “Much/Very” on question “How much urine leakage do you experience?”. We also evaluated urinary incontinence based on pad-use defined as 1 or more pads per 24 hours on question “How many pads do you use per 24 hours due to urinary leakage?”.
- Urinary urgency: defined as “Moderately” or “Much/Very” on question” Do you experience urinary urgency?”.
- Urinary obstructive symptoms: Urinary obstructive was defined as “Moderately” or “Much/Very” on question “Is your urine stream weak?”.
- Urinary bother: defined as “It would bother me moderately” or “It would bother me very much” on question: “If you were to live the rest of your life with your urinary tract function just as it is now, how would you experience this?”.

*Erectile function domain*

- Erectile dysfunction: defined as International Index of Erectile Function–5 score of less than 17 (e.g., “severe”, “moderate” and “mild to moderate” according to Cappelleri’s criteria). Patients using intracavernosal injections or intraurethral applications were also considered to have erectile dysfunction.
- Erectile bother: defined as answering “It would bother me moderately” or “It would bother me very much” on question “If you were to live the rest of your life with your sexual function just as it now is, how would you experience this?”.

*Quality of life domain*

- Global Quality of life: assessed by the question “How would you describe your quality of life?” rated on a Likert scale graded from 1 to 7 where 1-4 was defined as poor quality of life.
- Impact of treatment on daily activities: assessed by asking “How much does your prostate cancer illness or treatment affect your daily activities?” where “Moderately”, “Much/Very” was defined as a large impact on daily activities.

All the outcomes of the different domains were dichotomized to calculate absolute proportions.

**Urinary function**

| 4. How many pads do you use per 24 hours due to urinary leakage | □ | I do not use pads |
| --- | --- | --- |
|  | □ | Less than 1 per 24 hours |
|  | □ | Approximately 1 per 24 hours |
|  | □ | Approximately 2 per 24 hours |

|  | Not at all | A little | Moderately | Much/Very |
| --- | --- | --- | --- | --- |
| 1. How much urine leakage do you experience? | □ | □ | □ | □ |
| 2. Do you experience urinary urgency? | □ | □ | □ | □ |
| 3. Is your urine stream weak? | □ | □ | □ | □ |

| 5. If you were to live the rest of your life with your urinary tract function just as it is now, how would you experience this? | □ | It would not bother me at all |
| --- | --- | --- |
|  | □ | It would bother me a little |
|  | □ | It would bother me moderately |
|  | □ | It would bother me very much |

**Erectile function**

| 1. Have you used some kind of potency restoration method for sexual activity? | □ | No |
| --- | --- | --- |
|  | □ | Yes, self-injection treatment (e.g. Caverject) |
|  | □ | Yes, substance inserted into the urethra (e.g. Bondil) |
|  | □ | Yes, pills (e.g. Viagra, Cialis, Levitra) |
|  | □ | Yes, vacuum pump |
|  | □ | Yes, other |

|  | Very weak or  non-existent | Weak | Median | Strong | Very strong |
| --- | --- | --- | --- | --- | --- |
| 2. How would you asses your faith in getting and keeping an erection the past month? | □  1 | □  2 | □  3 | □  4 | □  5 |

| 3. How often after sexual stimulation has your erection, during the past month, been enough for penetration? | No sexual activity has occurred | Never or almost never | Less than half of the times | Half of the times | More than half of the times | Almost always or always |
| --- | --- | --- | --- | --- | --- | --- |
|  | □  0 | □  1 | □  2 | □  3 | □  4 | □  5 |

| 4. How often have you, during intercourse, been able to keep your erection after penetration the past month? | No attempts of intercourse have occurred | Almost never or never | Less than half of the times | Half of the times | More than half of the times | Almost always or always |
| --- | --- | --- | --- | --- | --- | --- |
|  | □  0 | □  1 | □  2 | □  3 | □  4 | □  5 |

| 5. How difficult have you found it to keep your erection until the end of the intercourse the past month? | No attempts of intercourse have occurred | Very great difficulties | Great difficulties | Difficult | Some difficulties | No difficulties |
| --- | --- | --- | --- | --- | --- | --- |
|  | □  0 | □  1 | □  2 | □  3 | □  4 | □  5 |

| SATISFACTION  6. When you have tried to have intercourse in the past month, how often have you experienced it as satisfying? | No attempts of intercourse have occurred | Almost never or never | Less than half of the times | Half of the times | More than half of the times | Almost always or always |
| --- | --- | --- | --- | --- | --- | --- |
|  | □  0 | □  1 | □  2 | □  3 | □  4 | □  5 |

| 7. If you were to live the rest of your life with your sexual function just as it now is, how would you experience this? | □ | It would not bother me at all |
| --- | --- | --- |
|  | □ | It would bother me a little |
|  | □ | It would bother me moderately |
|  | □ | It would bother me very much |

**Global Quality of Life**

| 1. How would you describe your quality of life? | Very poor | □  1 | □  2 | □  3 | □  4 | □  5 | □  6 | □  7 | Excellent |
| --- | --- | --- | --- | --- | --- | --- | --- | --- | --- |

|  | Not at all | A little | Moderately | Much/Very |
| --- | --- | --- | --- | --- |
| 2. How much does your prostate cancer illness or treatment affect your daily activities? | □ | □ | □ | □ |

**Supplementary table 1.** Multivariable logistic regression analysis after inverse probability of treatment weighting of 1455 men who had filled baseline and 1-year ePROM after robot-assisted radical prostatectomy and had complete data on baseline erectile function.

The model was adjusted for: age at surgery, multidimensional comorbidity index and Drug comorbidity index at time of surgery, pathological Gleason score and T stage, preoperative prostate-specific antigen, prostate volume at diagnosis, educational level and civil status. In addition, baseline erectile function was included.

| Prostatectomy type | Unweighted | Weighted |
| --- | --- | --- |
|  | Erectile dysfunction | |
| Primary prostatectomy | Ref (1.00) | Ref (1.00) |
| Delayed prostatectomy | 0.98 (0.85 to 1.13) | 0.95 (0.82 to 1.09) |
|  | Erectile bother | |
| Primary prostatectomy | Ref (1.00) | Ref (1.00) |
| Delayed prostatectomy | 0.96 (0.81 to 1.15) | 0.99 (0.82 to 1.20) |

**Supplementary table 2.** Characteristics of responders vs. non-responders to the 1-year ePROM questionnaire after primary or delayed robot-assisted radical prostatectomy.

1. At diagnosis

| **Variable** | **Primary prostatectomy No. (%)** | | **Delayed prostatectomy No. (%)** | |
| --- | --- | --- | --- | --- |
|  | **Responders** | **Non-responders** | **Responders** | **Non-responders** |
| No. of patients | 2751 (100) | 1604 (100) | 921 (100) | 390 (100) |
| **Age, years** |  |  |  |  |
| Median  [Interquartile range (IQR)] | 64 (59 - 69) | 63 (58 - 69) | 63 (59 - 67) | 64 (59 - 68) |
| <60 years | 728 (26) | 491(31) | 272 (30) | 110 (28) |
| 60-64 years | 661 (24) | 398 (25) | 264 (29) | 98 (25) |
| 65-70 years | 788 (29) | 385 (24) | 284 (31) | 126 (32) |
| 71-75 years | 574 (21) | 330 (21) | 101 (11) | 56 (14) |
| **Educational level*** |  |  |  |  |
| High | 1,138 (41) | 499 (31) | 388 (42) | 113 (29) |
| Intermediate | 1,202 (44) | 736 (46) | 378 (41) | 170 (44) |
| Low | 404 (15) | 351 (22) | 154 (17) | 104 (27) |
| Missing | 7 | 18 | 1 | 3 |
| **Civil status** |  |  |  |  |
| Married | 2,354 (86) | 1,280 (80) | 763 (83) | 312 (80) |
| Divorced | 340 (12) | 281 (18) | 135 (15) | 63 (16) |
| Widower | 57 (2) | 43 (3) | 23 (2) | 14 (4) |
| Missing | 0 | 0 | 0 | 1 |
| **Life expectancy** |  |  |  |  |
| Median (IQR) | 20 (17 - 25) | 21 (17 - 25) | 21 (18 - 25) | 20 (17–25) |
| **PSA (ng/mL)** |  |  |  |  |
| Median (IQR) | 5.7 (4.1 - 8.1) | 6.3 (4.5 - 9.0) | 5.2 (4.0 - 7.0) | 5.6 (4.4 - 7.5) |
| **Prostate volume (cc)** |  |  |  |  |
| Median (IQR) | 35 (28 - 46) | 35 (27 - 45) | 36 (29 - 48) | 37 (30 - 48) |
| Missing | 68 | 36 | 31 | 15 |
| **PSA density (ng/mL/cc)** |  |  |  |  |
| Median (IQR) | 0.16 (0.11 - 0.23) | 0.17 (0.13 - 0.25) | 0.14 (0.11 - 0.19) | 0.15 (0.11 - 0.20) |
| Missing | 68 | 36 | 31 | 15 |
| **Gleason score at diagnostic biopsy** |  |  |  |  |
| Gleason 6 | 654 (24) | 377 (24) | 821 (89) | 352 (90) |
| Gleason 7 (3+4) | 2,097 (76) | 1,227 (76) | 100 (11) | 38 (10) |
| **Clinical T stage** |  |  |  |  |
| T1 | 1,883 (68) | 1,071 (67) | 794 (86) | 336 (86) |
| T2 | 868 (32) | 533 (33) | 125 (14) | 53 (14) |
| Missing | 2 | 0 | 0 | 1 |

*Low: less than 10 years (mandatory school), intermediate: 10–12 years (high school), high: more than 12 years of education (university)

1. At RARP

|  | **Primary prostatectomy No. (%)** | | **Delayed prostatectomy No. (%)** | |
| --- | --- | --- | --- | --- |
| **Variable** | **Responders** | **Non-responders** | **Responders** | **Non-responders** |
| No. of patients | 2751 (100) | 1604 (100) | 921 (100) | 390 (100) |
| **Age** |  |  |  |  |
| Median (IQR) | 65 (60 - 69) | 64 (59 - 69) | 67 (62 - 71) | 68 (63 - 72) |
| <60 years | 675 (25) | 462 (29) | 122 (13) | 50 (13) |
| 60-64 years | 651 (24) | 392 (24) | 219 (24) | 81 (21) |
| 65-70 years | 768 (28) | 386 (24) | 256 (28) | 118 (30) |
| 71-75 years | 657 (24) | 364 (23) | 324 (35) | 141 (36) |
| **PSA (ng/mL)** |  |  |  |  |
| Median (IQR) | 5.9 (4.2 - 8.5) | 6.3 (4.5 - 9.2) | 7.1 (4.9 - 10.0) | 7.7 (5.5 - 11.0) |
| Missing | 5 | 4 | 17 | 3 |
| **Gleason score in prostatectomy specimen** |  |  |  |  |
| Gleason 6 | 500 (18) | 305 (19) | 404 (46) | 164 (43) |
| Gleason 7 (3+4) | 1,750 (64) | 995 (63) | 327 (37) | 139 (37) |
| Gleason 7 (4+3) | 453 (17) | 274 (17) | 123 (14) | 63 (17) |
| Gleason 8-9-10 | 14 (1) | 9 (1) | 31 (3) | 13 (3) |
| Missing | 34 | 21 | 36 | 11 |
| **Pathological T stage** |  |  |  |  |
| T2 | 1979 (73) | 1,101 (70) | 639 (70) | 251 (65) |
| T3 | 739 (22) | 468 (30) | 269 (30) | 137 (35) |
| Missing | 33 | 35 | 13 | 2 |
| **Time from diagnosis to RP, months** |  |  |  |  |
| Median (IQR) | 3 (2 - 5) | 3 (2 - 5) | 39 (24 - 57) | 37 (22 - 55) |
| ≤ 12 | 2517 (100) | 1,604 (100) | - | - |
| 13-24 | - | - | 230 (25) | 117 (30) |
| 25-48 | - | - | 352 (38) | 144 (37) |
| 49-72 | - | - | 228 (25) | 88 (23) |
| >72 | - | - | 111 (12) | 41 (11) |
| **Nerve sparing RARP** |  |  |  |  |
| No | 308 (11) | 222 (14) | 120 (13) | 56 (14) |
| unilateral | 625 (23) | 365 (23) | 179 (19) | 93 (24) |
| bilateral | 1,818 (66) | 1,017 (63) | 622 (68) | 241 (62) |
| **Surgical margins** |  |  |  |  |
| Negative | 2007 (74) | 1,127 (71) | 675 (74) | 249 (64) |
| Positive | 711 (26) | 459 (39) | 233 (25) | 138 (36) |
| Unclear | 12 (0) | 1 (0) | 4 (0) | 2 (1) |
| Missing | 21 | 17 | 9 | 1 |

**Supplementary Figure 1.** Flow chart of study population in Prostate Cancer data Base Sweden 5.0 of men with prostate cancer who according to the National Prostate Cancer Register underwent primary or delayed robot-assisted radical prostatectomy after a period of active surveillance from 2018 to 2020.
